# Predictions of Apoptosis Proteins by Integrating Different Features Based on Improving Pseudo-Position-Specific Scoring Matrix

**DOI:** 10.1155/2020/4071508

**Published:** 2020-01-14

**Authors:** Xiaoli Ruan, Dongming Zhou, Rencan Nie, Yanbu Guo

**Affiliations:** School of Information Science and Engineering, Yunnan University, Kunming 650504, China

## Abstract

Apoptosis proteins are strongly related to many diseases and play an indispensable role in maintaining the dynamic balance between cell death and division *in vivo*. Obtaining localization information on apoptosis proteins is necessary in understanding their function. To date, few researchers have focused on the problem of apoptosis data imbalance before classification, while this data imbalance is prone to misclassification. Therefore, in this work, we introduce a method to resolve this problem and to enhance prediction accuracy. Firstly, the features of the protein sequence are captured by combining Improving Pseudo-Position-Specific Scoring Matrix (IM-Psepssm) with the Bidirectional Correlation Coefficient (Bid-CC) algorithm from position-specific scoring matrix. Secondly, different features of fusion and resampling strategies are used to reduce the impact of imbalance on apoptosis protein datasets. Finally, the eigenvector adopts the Support Vector Machine (SVM) to the training classification model, and the prediction accuracy is evaluated by jackknife cross-validation tests. The experimental results indicate that, under the same feature vector, adopting resampling methods remarkably boosts many significant indicators in the unsampling method for predicting the localization of apoptosis proteins in the ZD98, ZW225, and CL317 databases. Additionally, we also present new user-friendly local software for readers to apply; the codes and software can be freely accessed at https://github.com/ruanxiaoli/Im-Psepssm.

## 1. Introduction

The location of proteins in organisms is closely related to their function and disease can occur following deviations in protein location [1]. The prediction of apoptosis protein localization began in 2003; this is a significant part of proteomics and one of the hotspots of bioinformatics [2]. The use of apoptosis proteins is a key by which organisms maintain homeostasis. Normally, cells maintain a balance between increasing proliferation and apoptosis, but too much or too little apoptosis can lead to many diseases [3, 4]. Cancer and AIDS are currently the most serious diseases threatening human health, and are linked with insufficient apoptosis and excessive apoptosis, respectively. Nowadays, predictive performance falls short of researchers' expectations that utilize crystal structures with high X-ra and NMR to analyze and calculate massive biological information datasets [5–7]. The distinct advantage of using machine learning to construct prediction models, compared with the tradition methods, are their high precision, low cost, and objectivity, due to the experiment reproducibility and the few subjective factors used during calculation. Therefore, research into the locations of apoptosis proteins could positively enrich the cognition of disease mechanisms and also further push forward the development of new drugs [8].

Currently, there are many research application machines learning to improve protein predictive accuracy, which have received major attention for their improved feature expression and optimization of the classification model. The feature expression of protein sequences principally includes the following five aspects. (i) Prediction of N-terminal information. Nakai and Kanehisa first adopted the N-terminal sorting signal to predict protein subcellular location in 1991 [9]. However, this method relied heavily on the assigned quality of the 5′ terminal of the gene or the *N*-terminal sequence of the protein, and this quality of assignment was often unreliable [10]. (ii) Prediction of Amino Acid Composition (AAC), such as ACC_GA [11], ACC_RPF [12]. ACC was first introduced by Nakashima [12]; this only calculated the frequency information of 20 amino acids, but easily resulted in the loss of rich information due to ill-considered ordering of amino acids in the sequence. (iii) Prediction of pseudo amino acid composition (Pseacc). Pseacc [13] overcomes the shortcomings of the ACC method by considering not only the sequence information of the original sequence, but also the physical and chemical properties of the protein. Similar methods include Pseacc_KPACP [14], Markov chains [15], and OA_Pseacc [16]. (iv) Prediction of evolutionary information of protein sequences. The evolutionary information of protein sequence is obtained by analyzing sequence homology, and some published articles revealed that this could achieve better performance in protein prediction. Methods include the Position Specific Scoring Matrix (PSSM) [17], Psepssm [2], and Gene ontology (Go) [4]. (v) Fusion of multiple feature expression. This has been regarded as one of the most prevalent methods, in which the core was to overcome the limitations of single feature expression and enrich the expression of features [18–20]. Additionally, there are some frequently used classification models such as the adaboost algorithm [21], Bayesian classifier [22], Fuzzy *K*-Nearest Neighbor (FKNN) algorithm [23], and SVM [16]. Among these, the SVM algorithm is one of the most widely used and effective classifiers in the field of biological information.

**Figure 1:**
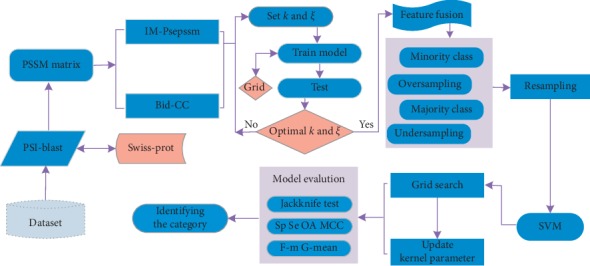
Framework of the proposed prediction model.

Some researches stated clearly that their use of evolutionary information of proteins for feature expression showed powerful performance; however, these methods still have flaws and leave room for improvement. Firstly, it is difficult to obtain both more effective feature expression and lower feature dimension based on the PSSM matrix without using the dimensionality reduction algorithm. Secondly, the existing webservers immensely limit the maximum number of protein sequences per processing; the majority of these merely yield predicted results, without providing extraction of relevant method features. Finally, class imbalance still exists. How to construct a predictive model with high prediction precision has become core research content in this field [24]. Therefore, in this paper, to overcome the above disadvantages and to improve the prediction accuracy of apoptosis proteins, the IM-Psepssm algorithm and bidirectional correlation coefficient algorithm were incorporated into the feature expression of the protein sequence, and then the resampling method of oversampling and undersampling method were employed to address the problem of data imbalance. Finally, the parameters of the SVM classifier were optimized by the grid search method to the classification prediction. Additionally, we present friendly local software for readers, which not only unlimits the scale per proceeding, but also provides download function for generating features of paper method for users. We used the sensitivity, specificity, Matthew correlation coefficient, *F*-measure, *G*-mean, and OA as evaluation indexes and applied the jackknife test method to verify the method on the ZD98, CL317, and ZW225 datasets. Our experimental results indicated that the proposed method remarkably improves significant indicators of apoptosis protein prediction. The framework of this proposed prediction model is shown in Figure 1.

## 2. Materials and Methods

### 2.1. Datasets

It is crucial to choose a representative and objective dataset to evaluate the performance of the prediction model. In our work, three different datasets; ZD98, ZW225, and CL317 were selected for comparison with other related methods. All of the protein sequences in the three datasets were extracted from SWISS-PROT (http://www.ebi.ac.uk/swissprot/), and the accession numbers of the protein sequences can be found from [25]. The distribution of the sequence identity percentage is as follows: sequence identity ≤40% occupies 34.69%, sequence identity from 41% to 80% occupies 30.6%, sequence identity from 81% to 90% occupies 17.35%, and sequence identity ≥91% occupies 17.35%. The CL317 dataset consists of six subcellular locations, and the datasets of ZD98 and ZW225 have four subcellular locations. More details on these datasets are shown in Table 1.

### 2.2. Position-Specific Scoring Matrix (PSSM)

PSSM [17] is one type of popular feature expression which contains rich information on the evolution of protein sequences. The evolutionary information of proteins makes a difference for the structure and function of protein sequences to same degree. The matrix of PSSM can be obtained using the PSL-BLAST program [26] to contrast protein sequences of the datasets (ZD98, ZW225, and CL317) and the nonredundant (NR) database (ftp://ftp.ncbi.nih.gov/blast/db/). In our work, the iterative times and the threshold of PSL-BLAST program were set 3 and 0.001, respectively. Each amino acid in the sequence can be given a specific fraction, and the PSSM of each protein sequence can be represented by equation (1):

(1) $$\begin{array}{c} P_{\text{PSSM}}=\left[\begin{array}{cccccc} M_{1,1} & M_{1,2} & \cdots & M_{1,j} & \cdots & M_{1,20}\\ M_{2,1} & M_{2,2} & \cdots & M_{2,j} & \cdots & M_{2,20}\\ \vdots & \vdots & \vdots & \vdots & \vdots & \vdots \\ M_{i,1} & M_{i,2} & \cdots & M_{i,j} & \cdots & M_{i,20}\\ \vdots & \vdots & \vdots & \vdots & \vdots & \vdots \\ M_{L,1} & M_{L,2} & \cdots & M_{L,j} & \cdots & M_{L,20} \end{array}\right]. \end{array}$$

The PSSM matrix is *L* × 20 for each protein sequence, where *L* expresses the length of each protein sequence, the 20 columns refer to the kinds of amino acids, and *M* represents the position specific score of amino acids such that the *i*-th position along the protein sequence is mutated to the *j*-th position. The sigmoid function scales the elements in the PSSM matrix to range from 0 to 1, in order to cut down the noise and bias, and the sigmoid function can be represented as follows:

(2) $$\begin{array}{c} f\left(x\right)=\frac{1}{1+e^{-x}}, \end{array}$$

where *x* is the element of PSSM matrix.

### 2.3. Improved Pseudo-Position-Specific Scoring Matrix (IM-Psepssm)

**Figure 2:**
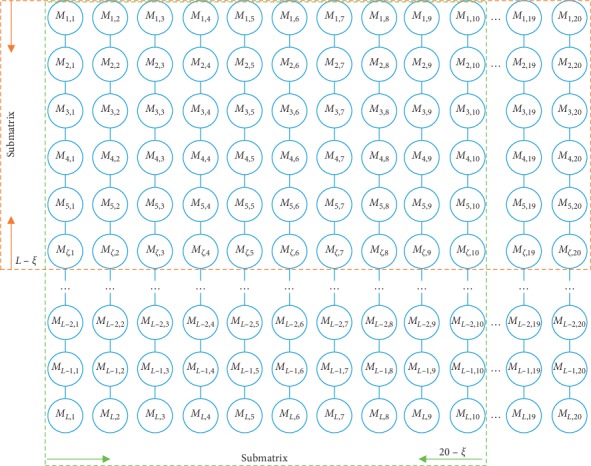
The segment of submatrix for Psepssm and IM-Psepssm.

Psepssm [27] is a feature expression based on the PSSM, which not only varies lengths of PSSM matrix to the uniform, but also considers the biological information and position information of residues in the sequence. However, it primarily concentrates on the local of the same column and loses some useful discriminatory information in different columns. In addition, the feature dimension increases with the distance parameter *ξ* gets larger. Therefore, an improved method of feature extraction is proposed based on PSSM, namely, feature extraction from different columns on the premise of dimension reduction. The IM-Psepssm is described as follows:

- Segment the PSSM. The PSSM matrix of equation (2) in divided into submatrices of different sizes according to different parameters, *ξ*. The research is commonly divided by row, and the size of a submatrix is usually (*L* − *ξ*, 20). Quite a few protein sequences are commonly long (*L* > 20), and with the increase of the parameter *ξ*, many effective features will be lost. But the submatrix of the IM-Psepssm approach is segmented by column, namely (*L*, 20 − *ξ*), and it will preserve more rich features. The submatrix is shown in Figure 2, and the green region is the IM-Psepssm submatrix, the orange is the Psepssm submatrix.
- Compute local features. It also differs from the traditional Psepssm algorithm in that it computes the cross-correlation factor of different types of amino acids rather than same property amino acids, and it only measures 20 − *ξ* amino acid pairs, so, the dimension of the feature vector for each protein sequence is (20 + 20 × *ξ*) − *ξ* × (*ξ* + 1)/2. This method allows for helpful dimension reduction without missing features. In our work, the value of *ξ* is determined by the highest predictive accuracy for different datasets. The calculation for each submatrix is as follows:

(3) $$\begin{array}{c} \text{IM}-P_{\text{Psepssm}}^{\xi }={\left[\bar{M_{1}},\bar{M_{2}},\ldots ,\bar{M_{20}},T_{1}^{\xi },T_{2}^{\xi },\ldots ,T_{20}^{\xi }\right]}^{T},\\ T_{n}^{\xi }=\frac{1}{L-\xi }\overset{20-\xi }{\underset{n=1}{\sum }}{\left[M_{m,n}-M_{m,\left(n+\xi \right)}\right]}^{2},\;m=1,2,\ldots ,L,\xi <n,\xi \neq 0,\\ \bar{M_{j}}=\frac{1}{L}\overset{L}{\underset{i=1}{\sum }}M_{i,j},\;j=1,2,\ldots ,20, \end{array}$$

where *T* denotes the transpose operator, *L* is the length of protein sequence, *n* represents 20 amino acids, and $\bar{M_{j}}$ represents the average score of the amino acid residues in the protein *P* which are mutated to *j-*th type amino acid during the evolution process. To avoid the loss of sequence-order information, the order factor *T*_*n*_^*ξ*^ is added. In addition, some methods based on the IM-Psepssm had been tested in the experimental parts of this paper.

(4) $$\begin{array}{c} {T^{1}}_{n}^{\xi }=\frac{1}{L-\xi }\overset{20-\xi }{\underset{n=1}{\sum }}M_{m,n}*M_{m,\left(n+\xi \right)},\;\left(m=1,2,\ldots ,L,\xi <n,\xi \neq 0\right), \end{array}$$

(5) $$\begin{array}{c} \left\{\begin{array}{c} R_{k}=\sum_{i=1}^{k}m_{i},\;k=1,2,\ldots ,L,\\ S_{k}=\sum_{i=1}^{c}n_{i},\;c=1,2,\ldots ,20, \end{array}\right. \end{array}$$

(6) $$\begin{array}{c} {T^{2}}_{n}^{\xi }=\frac{1}{L-\xi }\overset{20-\xi }{\underset{n=1}{\sum }}\left(M_{m,n}-{\tilde{R}}_{k}\right)*\left(M_{m,n+\xi }-{\tilde{R}}_{k}\right),\;\left(m=1,2,\ldots ,L,\xi <n,\xi \neq 0\right), \end{array}$$

(7) $$\begin{array}{c} {T^{3}}_{n}^{\xi }=\frac{1}{L-\xi }\overset{20-\xi }{\underset{n=1}{\sum }}\left(M_{m,n}-{\tilde{S}}_{k}\right)*\left(M_{m,n+\xi }-{\tilde{S}}_{k}\right),\;\left(m=1,2,\ldots ,L,\xi <n,\xi \neq 0\right), \end{array}$$

where {*R*_*k*_} and {*S*_*t*_} represent the sum for different rows and columns in PSSM matrix, ${\tilde{R}}_{k}$ and ${\tilde{S}}_{k}$ are the mean value of *R*_*k*_ and *S*_*k*_, respectively.

### 2.4. Bidirectional Correlation Coefficient Algorithm

The idea of Bid-CC [28] was first proposed by Harsh Saini et al. in 2016. This method chiefly describes the relationships between two residues in different positions along the protein sequence. Every different value of *S* will generate 400 different feature vectors, where *S* represents the distance between amino acid pairs. The setting of *S* value is decided by the classification performance of different datasets. In addition, to reduce the redundant information of feature vectors and reduce the dimensions of data, the dimension of feature vectors correspond to the single value of *S* with high prediction accuracy rather than 400 ∗ *S*. The equation for this method is given below:

(8) $$\begin{array}{c} T_{m,n}\left(S\right)=\overset{L-S}{\underset{i=1}{\sum }}M_{i,m}M_{i+S,n},\;\left(1\leq m\leq 20;\;1\leq n\leq 20;\;S<L\right),\\ T\left[S\right]=\left[T1,1\left[S\right],T1,2\left[S\right],\ldots ,T1,20\left[S\right],\;T2,1\left[S\right],\;T2,2\left[S\right],\ldots ,T2,20\left[S\right],\ldots ,T20,1\left[S\right],\ldots ,T20,20\left[S\right]\right], \end{array}$$

where *m* and *n* represent the 20 different amino acids, *L* is the length of protein sequence, and *S* is the distance between amino acid pars.

### 2.5. Resampling Method

The algorithm prediction performance is impacted by the unbalanced data distribution. Synthetic minority oversampling technique (SMOTE) is a better oversampling method to deal with the problem of data imbalance [29], which overcomes the problem of overfitting caused by the random sampling method only copying minority class samples. The idea of this method is to artificially synthesize new samples and construct a new balanced dataset by analyzing minority class samples and linearly interpolating in the minority class samples neighborhoods. However, the traditional SMOTE method did not consider the distribution characteristics of neighboring samples and might cause duplication between categories [30]. The classical undersampling method ENN could effectively remove redundant and noisy samples sandwiched in minority class samples. Therefore, the SMOTEENN method is adopted to solve the problem of sample imbalance, which gives the model strong generalization ability. The steps of the algorithm are as follows:

  Step 1. For each minority sample *x* in the dataset, *k* nearest neighbor samples of the same class are selected by calculating Euclidean distance.
  Step 2. According to the oversampling magnification *N*, *N* × *k* samples are randomly selected from *k* neighbor samples from Step 1, namely, *y*_1_, *y*_2_,…, *y*_*N*×*k*_.
  Step 3. Each sample *x* and *y*_*i*_ are randomly interpolated to generate a new sample *x*_new_, namely,

(9) $$\begin{array}{c} x_{\text{new}}=x+\text{rand}\left(0,1\right)\times \left(y_{i}-x\right),\;i=1,2,\ldots ,N\times k, \end{array}$$

  where rand (0, 1) represents the random number between [0, 1].
  Step 4. *x*_new_ generated by Step 3 is added to the original dataset and a new *X*_new_ is obtained.
  Step 5. Traverse each sample *x*_*i*_ in *X*_new_ and find three neighbor samples of each *x*_*i*_. If two or more of the three nearest neighbor samples are different from the class of the sample, the sample *x*_*i*_ is deleted.
  Step 6. Update *X*_new_ and use classifier to learn it.

### 2.6. Support Vector Machine

SVM [31, 32] is a classical classification algorithm that can perform global optimization and prevent overfitting. In recent years, it has been widely applied in bioinformatics research to solve various classification and prediction problems. The selection of kernel function and the setting of kernel parameters are significant parts for the SVM model. In this paper, we selected radial basis function as kernel function due to it is superiority in dealing with the problem of nonlinearity. The parameters of cost (*c*) and the kernel width (*γ*) were optimized by the grid search method on the training dataset. More attention was paid to the total error in the whole optimization process with the *c* being larger, so the prediction accuracy was higher in the training samples. In contrast, some misclassification samples will be allowed in the training samples, and the model will have a stronger generalization ability with the *c* reduced. In the case of training samples with noise, the latter is generally used, and the incorrectly classified samples in the training samples set are considered noise.

### 2.7. Performance Evaluation and Validation Method

There are several frequently used validation methods to measure the performance of the prediction model in statistical prediction, including the jackknife test, independent dataset test, and k-fold cross validation [33]. Due to jackknife test's advantage for small sample datasets and the predicted result unique after many repeated experiments to the same dataset [34, 35], this method was chosen to examine the performance of our method. Furthermore, to comprehensively evaluate the reliability and effectiveness of the proposed method, we adopt the following six standard evaluation indicators for measurement: Sensitivity (Sn), Specificity (Sp), *F*-measure (*F*-m), Overall Accuracy (OA), Mathew's correlation coefficient (Mcc), and *G*-mean, which are shown as follows:

(10) $$\begin{array}{c} \text{Recall or Sn}\left(i\right)=\frac{\text{TP}}{\text{TP}+\text{FN}},\\ \text{Sp}\left(i\right)=\frac{\text{TN}}{\text{TN}+\text{FP}},\\ F-\text{measure}=\frac{\text{Precision}\times \text{Recall}}{\text{Precision}+\text{Recall}}\times 2,\\ \text{OA}=\frac{\text{TP}+\text{TN}}{\text{TP}+\text{FN}+\text{FP}+\text{TN}},\\ \text{MCC}\left(i\right)=\frac{\text{TP}\times \text{TN}-\text{FP}\times \text{FN}}{\sqrt{\left(\text{TP}+\text{FP}\right)}\left(\text{TP}+\text{FN}\right)\left(\text{TN}+\text{FP}\right)\left(\text{TN}+\text{FN}\right)},\\ G-\text{mean}=\sqrt{\frac{\text{TP}}{\text{TP}+\text{FN}}\times \frac{\text{TN}}{\text{TN}+\text{FP}}}, \end{array}$$

where FP, FN, TP, and TN are the number of false positives, false negatives, true positives, and true negatives, respectively. The *Se* represents the prediction accuracy of each category. If Se is higher and Sp is lower; it indicates that a higher rate of false positives is generated in the actual prediction. If Sp is high and Se is low, a high percentage of false negatives will occur in the actual prediction. *G*-mean and *F*-m are the primary evaluation indicators for classification of unbalanced datasets, the higher the value is, the better the handling of data imbalance issues. The source codes were written in the programming language Python 3.6.4 on PC with Intel i5 7400 3.00 GHz CPU, 16 GB RAM, and GTX1080ti GPU.

## 3. Result and Discussion

### 3.1. Selection of Optimal Parameters *ξ* and *S*

Single feature extraction strategy is hard to acquire rich discriminant information. In this paper, IM-Psepssm and the BID-CC algorithm are fused to feature expression. Compared with traditional Psepssm, the obvious advantage of IM-Psepssm is its lower dimension feature vectors under the same parameter *ξ* for feature extraction. The parameter *ξ* has a certain impact on the dimension of the feature vector, if the value of *ξ* is taken too large, excessive redundancy features will be added and the prediction performance of the algorithm will be affected, and if the value of *ξ* is taken too small, some key features in the protein sequence will be missed. Therefore, values in the range 0–15 comprehensively on account of the prediction results in three datasets.

**Figure 3:**
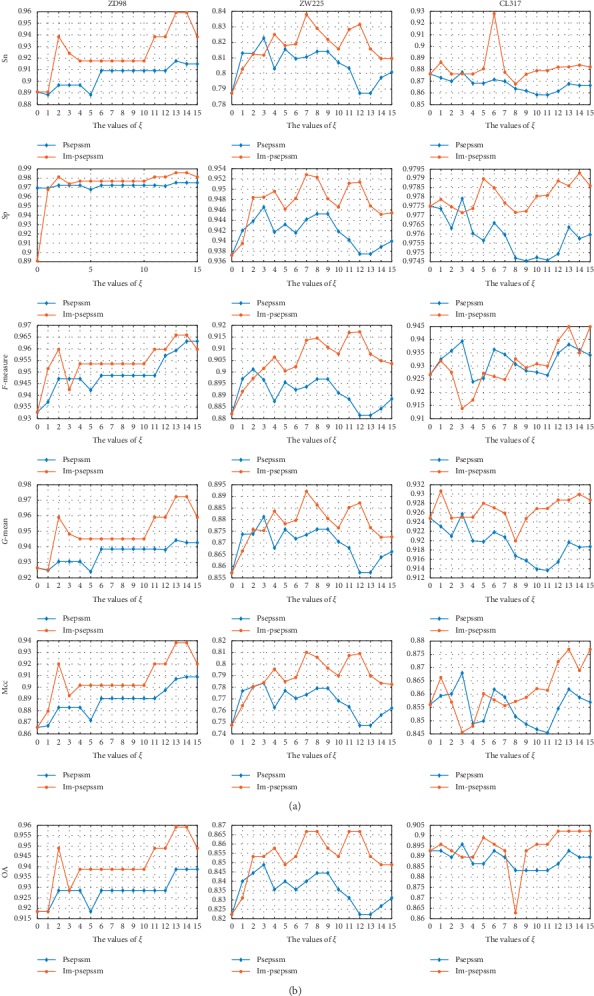
Effect of selecting different values of *ξ* on CL317, ZW225, and ZD98 datasets by jackknife test.

The feature expression methods of IM-Psepssm and Psepssm were used to conduct classification prediction using the SVM classifier and jackknife method under the different parameters *ξ*, and the comparison results are given in Figure 3. The overall prediction accuracy of the IM-Psepssm method was higher than Psepssm on the ZD98, ZW225, and CL317 datasets, respectively. Among them, for the CL317 dataset, the IM-Psepssm method achieved the highest prediction accuracy of 90.22% when *ξ* was set to 12 (182D), which is higher than that of the traditional Psepssm by 1.58% under the same *ξ* parameter. For the ZW225 dataset, the IM-Psepssm and Psepssm showed the highest prediction accuracy of 86.67% and 84.88%, respectively, when *ξ* was set to 7 (132D) and 3 (80D). The prediction accuracy of IM-Psepssm was 2.67% and 0.45% higher than that of Psepssm under the same *ξ* value. Additionally, on the ZD98 dataset, when *ξ* was set to 13 (189D) and 13 (280D), IM-Psepssm and Psepssm methods achieved the highest prediction accuracy of 95.92% and 93.87%, respectively, The accuracy of IM-Psepssm was 2.05% higher than that of Psepssm under the same *ξ*. Additionally, the other evaluation indicators of IM-Psepssm (such as Sp, Sn, Mcc, G-mean, and F-measure) were also superior to those of Psepssm under the same parameters. The description based on the above experimental results indicates that the IM-Psepssm method has a richer feature expression.

**Figure 4:**
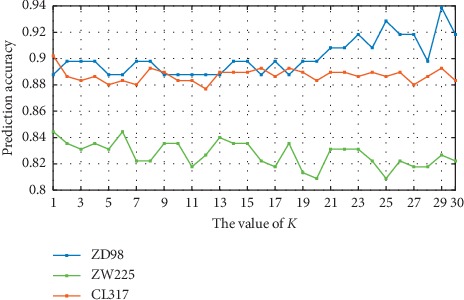
Effect of selecting different values of *k* on CL317, ZW225, and ZD98 datasets by jackknife test.

The BID-CC algorithm is another method used to transform PSSMs matrices with various lengths into fixed-length feature vectors from two different directions. The 400-dimensional eigenvectors will be generated for different parameters of *k*, and different *k* values contain different effective feature expressions.  Figure 4 reveals the variation trend of OA on three different datasets by setting different *k* values. The experimental results showed that the overall prediction accuracy of the ZW225 dataset was lower than those of the other two datasets because its homologous information is less; for example, the proportion of sequences with homology less than 40% is 52.9%. Secondly, with the experimental results of three datasets, the prediction accuracy basically tends to be stable and declines when *k* reaches 30, so the value of *k* should be kept in the range 0–30. Finally, the highest prediction accuracy of ZD98, ZW225, and CL317 datasets was 93.87%, 84.44%, and 90.22% when *k* was set to 29, 1, and 1, respectively. For the CL317 dataset, when the value of *k* changes, the fluctuation range of prediction accuracy becomes smaller than that of the ZD98 and ZW225 datasets, and when *k* is higher than 21, the fluctuation range decreases and becomes more stable for the ZW225 dataset.

### 3.2. Analysis of Different Feature Expression

The experimental results of Section 3.1 show that IM-Psepssm not only has better prediction performance than the traditional Psepssm algorithm, but also has lower feature dimensions under the same parameter *ξ*. Based on the gain of low-dimensional effective feature expression, this paper compares the other three feature expression methods proposed in this paper, which are T1-IM-PSSM, T2-IM-PSSM, and T3-IM-PSSM. Under the same parameter *ξ*, the experimental results show that the prediction accuracy is lower than IM-Psepssm. Among them, T3-IM-PSSM is the worst prediction effect, and the prediction accuracy of IM-Psepssm method is higher by 2.04%, 4%, and 0.64%; 1.02%, 5.33%, and 0; 5.1%, 6.6%, and 4.1% than T1-IM-PSSM, T2-IM-PSSM, and T3-IM-PSSM for ZD98, ZW225, and CL317, respectively. Secondly, apart from the CL317 dataset, the IM-Psepssm and the T2-IM-PSSM basically research the same prediction result, which implies that the T2-IM-PSSM is also a good feature expression method. Finally, the T2-IM-PSSM method is similar to the T3-IM-PSSM from equations (6) and (7), but the prediction result is higher by 4.08%, 1.33%, and 4.1% for the ZD98, ZW225, and CL317 datasets, respectively.

The feature expression methods of IM-Psepssm and BID-CC based on the PSSM matrix are complementary. Both methods are designed to extract richer apoptosis protein sequence features from the PSSM matrix. To compare the contribution of the two methods with the fusion feature BIM-PSSM, a comparison of the results of IM-Psepssm, BID-CC, and BIM-PSSM is also shown in Table 2. The best prediction accuracies of IM-Psepssm algorithm were achieved when the parameter *ξ* was 13, 7, and 12 on ZD98, ZW225, and CL317 datasets, respectively. The optimal prediction accuracies of the BID-CC algorithm were associated when the parameter *k* was set as 29, 1, and 1, respectively. The results reveal that the BIM-PSSM was higher than IM-Psepssm and BID-CC, but the OA of BIM-PSSM method was 2.04%, 3.11%, and 1.26% higher than BID-CC on ZD98, ZW225, and CL317, respectively. The OA of the IM-Psepssm method is 0.89% and 1.26% lower than the BIM-PSSM method on ZW225 and CL317, respectively, which indicates that both methods make a positive contribution but the IM-Psepssm method is more helpful for the fusion method. Additionally, the rest evaluation indicator of BIM-PSSM, Sn, Sp, *F*-m, Mcc, and *G*-mean is also better than that of IM-Psepssm, T1-IM-PSSM, T2-IM-PSSM, T3-IM-PSSM and BID-CC method, which indicates that feature fusion is positive for boosting the prediction performance.

### 3.3. Performance Comparison of Different Sampling Methods

**Figure 5:**
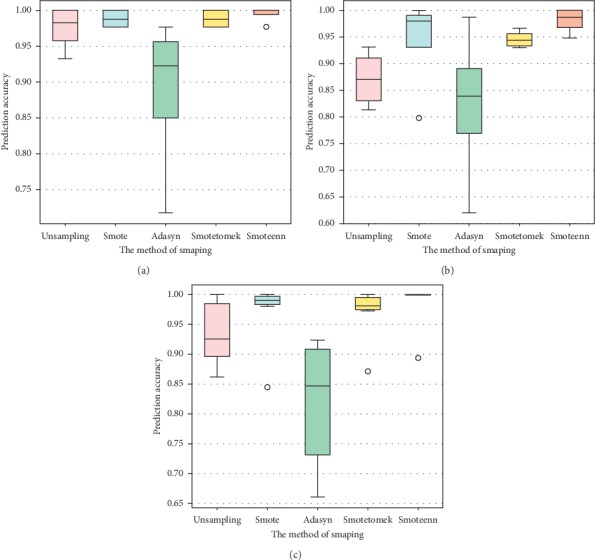
Comparison of sampling methods for each class of samples. (a) ZD98. (b) ZW225. (c) CL317.

The apoptosis protein datasets are unbalanced, as can be seen in Table 1. This will influence the prediction effect of the algorithm. To verify the validity of the sampling method, this paper contrasts the without-sampling method and the different sampling methods. The unsampling method refers to the best feature extraction method obtained by Table 2, and the sampling methods contain the SMOTE, ADASYN, SMOTEENN, and SMOTTETomek methods. We can see in Figure 5 that the OA for each class is 93.3–100%, 97.67–100%, 71.69–97.67%, 97.67–100%, and 97.72–100% on the ZD98 dataset for unsampling, using the SMOTE, ADASYN, SMOTEENN, and SMOTETomek methods, respectively. The OA for each class is 81.39–93.18%, 61.9–98.70%, 79.81–96.70%, 94.80–98.86%, and 94.80–100% on the ZW225 dataset for unsampling, using the SMOTE, ADASYN, SMOTEENN, and SMOTETomek methods, respectively. The OA for each class is 86.27–100%, 84.49–99.10%, 66.02–92.38%, 87.2–100%, and 89.43–100% on the CL317 dataset for unsampling, using the SMOTE, ADASYN, SMOTEENN, and SMOTETomek methods, respectively. Among them, the ADASYN is the worst and the average value is lower than that of the without-sampling method. Although the ADASYN and SMOTE methods both are popular sampling methods, ADASYN is more susceptible to outlier sample points. The resampling method of SMOTEENN basically achieves the optimal prediction result for three different apoptosis protein datasets, both are higher than the unsampling method and the SMOTE method, which indicates that the method not only removes duplicate samples but also has more stability in dealing with sample balance problems.

By comparing the sampling methods in Figure 5, the results show that the optimal prediction results are obtained through combination of oversampling and undersampling for processing protein class balance. To prove the power of the resampling method to improve apoptosis protein prediction, two different fusion and resampling strategies are compared. The strategies 1 firstly adopts a resampling algorithm to balance the feature of IM-Psepssm and BID-CC, respectively, then fuses them together, namely, Resampling 1. In the strategies 2, the IM-Psepssm and BID-CC are fused first, and then a resampling algorithm is used to eliminate imbalance of the fusion eigenvector, namely, Resampling 2. The SVM classifier and jackknife verification methods were adopted to compare the results of the original data, Resampling 1, and Resampling 2, and the Mcc, *F*-m, and *G-*mean were used as evaluation indicators.

The experimental results are shown in Table 3. Among them, the *F*-m and *G*-mean are significantly comprehensive indicators for evaluating unbalanced data. The results reveal that the *F*-m and *G*-mean of three datasets are raised after use of either fusion and resampling strategies. ZD98 is the smallest dataset, and its boost is inferior to those of the CL317 and ZW225 datasets. In the ZD98, CL317, and ZW225 datasets, compared with the original dataset, *F*-m and *G*-mean of Resampling 1 and Resampling 2 are on average increased by 0.61% and 2.33%, 1.67% and 3.01%; 3.12% and 5.58%, 3.73% and 6.15%; and 7.1% and 8.17%, 8.04% and 9.16%, respectively, which declare that the resampling method improves the protein prediction performance. Secondly, Mcc is one kind of indicator to evaluate the effectiveness of the classifier. Looking at the results of the three datasets, the average Mcc of Resampling 2 is raised to 98.88%, 98.5%, and 97.31% on ZD98, CL317, and ZW225, respectively. The higher the Mcc value is, the better the classification performance is. Thirdly, the average value of the evaluation index of Resampling 2 is higher than Resampling 1, which reveals that resampling 2 is more helpful for identifying the subcellular locations of apoptosis proteins.

### 3.4. Performance Comparison with Other Models

To objectively prove the reliability and superiority of our method, we adopt the identical jackknife test method to compare it with other algorithms following use on the same datasets. The class accuracy and the overall accuracy were used as the evaluation indicator. The experiment results are shown in Tables 4–6 on the ZD98, Cl317, and ZW225 datasets, respectively, and the optimal prediction accuracy is marked in bold. The comparison algorithms include OF-SVM [3], FTD-SVM [20]], BOW-SVM [6], GA_DCCA-SVM [36], OA-SVM [16], PSSMP [17], IACC-SVM [18], EN-FKNN [37], Dual-layer SVM [38], and OA-MLSC [29]. The DCCC-SVM method was proposed by Liang et al. [39], which mainly adopted use of detrended cross-correlation coefficients for feature expression (FE). Zhang and Jin proposed the OF-SVM method [3], which in principle made the *λ*-Order factor for FE. The FTD-SVM method was put forward by Liang and Zhang [20], which fused frequencies of triplet codons and detrended the forward moving-average cross-correlation analysis method for FE. BOW-SVM adopted bag of words for FE by Zhao and Zhang [6]. The GA_DCCA-SVM method was put forward by Liang et al. [36], which fused Geary autocorrelation and DCCA coefficient for FE. The OA-SVM method was proposed by Zhang and Duan [16], this primarily adopted the oversampling method to handle the data unbalance problem. The IACC-SVM method was proposed by Zhang and Liang [18], which combined integrating the auto-cross correlation method and PSSM to feature expression. The above algorithm all use SVM as the classifier.

The result shows that the OA of this paper is higher by 8.49%–2.59%, 13.27%–7.47%, and 9.47%–2.47% than the contrast algorithm on ZD98, ZW225, and CL317 datasets, respectively. The algorithm achieves the highest in all classes on the ZD98 dataset. Among the ZD98 dataset, the performance of Other class is the highest increasing to 4.1%, compared with the first-best (OA-MLSC [29]). For the Se class on the CL317 dataset, it is only lower than the OA-SVM algorithm and higher for all others; the remaining classes are basically obtained optimally. Secondly, the OA-SVM and the OA-MLSC methods also utilize sampling methods to solve the problem of imbalance of apoptosis datasets, and experimental results reveal that this paper is higher than the OA-SVM method 6.09%, 5.47%, and 2.47% on ZD98, ZW225, and CL317 dataset, respectively, and higher than the OA-MLSC method 2.59% and 3.67% on the ZD98 and CL317 dataset, respectively, which indicate that the resampling method is better than the sampling approach alone. Thirdly, for the ZD98, ZW225, and CL317 datasets, the fluctuation range of each-class prediction accuracy is 2.28%, 5.2%, and 5.31%, which are basically less than other algorithms, and the experimental results show that this method has better stability. Fourthly, PSSM is an effective evolutionary information feature expression method based on protein sequence, and the PSSMP [17], FTD-SVM [20], and GA_DCCA-SVM [36] are all based on the PSSM matrix for feature expression. The experiments on the ZD98, ZW225, and CL317 datasets show that the proposed method is superior to these methods by 6.39% and 7.97%; 8.49%, 12.37%, and 9.47%; 7.49%, 13.27%, and 9.47%, respectively. It shows that this method can extract more effective information hidden in the PSSM matrix and improve the accuracy of apoptosis protein prediction.

## 4. Conclusions

An approach for predicting apoptosis proteins, which differs from previous methods, is proposed in this paper. Firstly, the IM-Psepssm algorithm enriches feature extraction which the traditional Psepssm method lacks and also has lower dimensions under the same parameters. Secondly, the SMOTEENN method is adopted to resample the dataset, which not just keeps the data balance but eliminates noise features. Thirdly, the parameters of the SVM classifier are optimized by use of the grid search method. Finally, the establishment of the SD-Psepssm Builder, which, unlike existing servers, is unlimited in the number of protein sequences uploaded by users and is more useful and easier for users to obtain the feature expression of protein sequence for their research. Few would dispute that this method could significantly increase ability to obtain richer feature information and to enhance the prediction accuracy of apoptosis protein prediction; moreover, this method has better stability. However, it still has room for improvement in the future work. We hope and try to construct multilabel dataset of apoptosis proteins and capture other types of features extraction methods (such as physicochemical features and structural features) based on our framework to increase the diversity of features and implement the integrated multiclassifier to enhance the accuracy and robustness of apoptosis protein prediction.

## Figures and Tables

**Table 1:** Data and the distribution of the sequence identity percentage for apoptosis.

Datasets	≤40%	41%–80%	81%–90%	≥91%	Cy	Me	Mi	Nu	En	Se	Sum
CL317	40.1	15.5	18.9	25.6	112	55	34	52	47	17	317
ZW225	52.9	16	16	15.1	70	89	25	41	—	—	225
Dataset	≤40%	41%–80%	81%–90%	≥91%	Cy	Me	Mi	Others	—	—	Sum
ZD98	34.69	30.61	17.35	17.35	43	30	13	12	—	—	98

**Table 2:** The contribution of two feature submodels for the final overall accuracy (%).

Datasets	Index	IM-Psepssm	T1-IM-PSSM	T2-IM-PSSM	T3-IIM-PSSM	BID-CC	BIM-PSSM
ZD98	Sn	95.92	93.26	93.84	88.0	93	94.41
	Sp	98.59	97.85	98.14	96.49	97.60	98.27
	*F*-m	96.59	94.77	95.97	94.11	96.19	97.88
	Mcc	93.82	90.41	92.0	86.28	91.52	94.32
	*G*-mean	97.23	95.48	95.90	91.81	95.20	96.24
	OA	95.91	93.87	94.89	90.81	93.87	95.91

ZW225	Sn	83.80	79.67	78.03	75.87	81	84.55
	Sp	95.28	93.82	93.34	92.68	94.4	95.52
	*F*-m	91.36	88.24	87.39	87.24	89.32	90.34
	Mcc	81.00	75.27	73.36	71.81	77.36	80.69
	*G*-mean	89.21	86.29	85.11	83.55	87.29	89.79
	OA	86.66	82.66	81.33	80	84.44	87.55

CL317	Sn	88.21	88.64	89.25	81.64	87.54	88.84
	Sp	97.88	97.76	97.89	96.79	97.85	98
	*F*-m	93.97	92.91	93.93	93.09	93.80	95.42
	Mcc	87.22	86.38	87.66	82.18	86.71	88.88
	*G*-mean	92.87	93.06	93.44	88.56	92.45	93.22
	OA	90.22	89.58	90.22	86.12	90.22	91.48

**Table 3:** Performance comparison of original data and sampling methods for each class of sample.

Dataset location	Original dataset			Resampling 1			Resampling 2		
	Mcc	*F*-m	*G*-mean	Mcc	*F*-m	*G*-mean	Mcc	*F*-m	*G*-mean
*ZD98*									
Cy	91.83	93.94	96.1	95.17	97.56	97.57	97.3	100	97.7
Me	100	100	100	98.35	100	98.83	100	100	100
Mi	90.24	100	91.29	96.44	96.43	99.09	98.2	98.2	99.3
Others	95.2	97.59	97.59	98.33	100	98.8	100	100	100

*CL317*									
Cy	86.43	91.26	93.7	96.56	96.56	99.63	97.31	97.28	99.71
Me	94.95	98.7	96.58	98.86	99.43	99.43	100	100	100
Mi	92.51	94.62	97.22	99.14	99.41	99.9	98.83	98.83	99.79
Se	89.91	96.44	93.6	97.78	99.44	98.54	96.13	100	96.85
Nu	82.62	91.48	90.76	95.2	96.98	97.98	99.37	99.37	99.9
En	86.87	100	87.45	96.23	99.4	97.28	99.41	99.41	99.89

*ZW225*									
Cy	79.44	88.62	89.98	91.65	92.79	97.61	94.7	94.64	99.11
Me	89.75	94.37	93.85	97.85	97.85	99.53	98.9	98.9	99.76
Mi	78.83	92.03	86.52	97.4	99.13	98.54	99.15	100	99.42
Nu	76.62	86.35	88.81	94.69	100	96.18	96.47	100	97.5

**Table 4:** Performance comparison of different models on ZD98 dataset.

Methods	Prediction accuracy (%)				
	Cy	Me	Mi	Others	OA
OF-SVM [3]	97.7	86.3	92.3	66.7	90.8
FTD-SVM [20]	95.4	93.3	76.9	83.3	90.8
BOW-SVM [6]	97.7	92.9	76.9	83.3	91.7
GA_DCCA-SVM [36]	95.4	90.0	92.3	83.3	91.8
OA-SVM [16]	95.3	88.9	97.4	91.7	93.2
PSSMP [17]	95.3	93.3	84.6	91.7	92.9
OA-MLSC [29]	100	96.7	92.3	95.9	96.7
This paper	**100**	**97.72**	**100**	**100**	**99.29**

**Table 5:** Performance comparison of different models on the ZW225 dataset.

Methods	Prediction accuracy (%)				
	Cy	Me	Mi	Nu	OA
OF-SVM [3]	85.7	91.0	68.0	82.9	85.3
FTD-SVM [20]	88.6	93.3	64.0	75.6	85.3
GA_DCCA-SVM [36]	87.1	91.0	68.0	75.6	84.4
OA-SVM [16]	93.3	92.1	96.0	93.5	92.2
IACC-SVM [18]	88.6	92.1	64.0	75.6	84.9
EN-FKNN [37]	94.3	94.4	60.0	80.5	88.0
Dual-layer SVM [38]	91.4	94.4	76.0	78.1	88.4
This paper	**100**	**100**	**97.47**	**94.80**	**97.67**

**Table 6:** Performance comparison of different models on the CL317 dataset.

Methods	Prediction accuracy (%)						
	Cy	Me	Mi	Se	Nu	En	OA
OF-SVM [3]	94.6	90.9	76.5	92.2	86.5	93.6	89.6
FTD-SVM [20]	92.9	89.1	82.4	70.6	86.5	93.6	89.0
BOW-SVM [6]	94.6	87.3	82.4	82.4	84.3	91.5	89.2
GA_DCCA-SVM [36]	92.9	89.1	82.4	76.5	84.6	93.6	89.0
OA-SVM [16]	96.1	95.7	93.9	**98.0**	95.5	100	96.0
IACC-SVM [18]	96.4	94.5	82.4	76.5	80.8	93.6	90.5
PSSMP [17]	92.0	92.7	82.4	76.5	90.4	93.6	90.5
EN-FKNN [37]	98.2	83.6	79.4	82.4	90.4	97.9	91.5
OA-MLSC [29]	95.5	93.6	96.4	94.1	94.2	94.1	94.8
This paper	**100**	**100**	**100**	94.69	**97.03**	**100**	**98.47**

## Data Availability

The codes, original data, and software can be freely accessed at http://github.com/ruanxiaoli/Im-Psepssm.

## References

[1] Wei L., Liao M., Gao X., Wang J., Lin W. (2016). mGOF-loc: a novel ensemble learning method for human protein subcellular localization prediction. *Neurocomputing*.

[2] Yu B., Shan L., Qiu W. Y. (2018). Prediction of subcellular location of apoptosis proteins by incorporating PsePSSM and DCCA coefficient based on LFDA dimensionality reduction. *BMC Genomics*.

[3] Zhang S. L., Jin J. (2017). Prediction of protein subcellular localization by using *λ*-order factor and principal component analysis. *Letters in Organic Chemistry*.

[4] Zhong J., Sun Y., Peng W., Xie M., Yang J., Tang X. (2018). XGBFEMF: an XGBoost-based framework for essential protein prediction. *IEEE Transactions on NanoBioscience*.

[5] Borkowski O., Bricio C., Murgiano M. (2018). Cell-free prediction of protein expression costs for growing cells. *Nature Communications*.

[6] Zhao N., Zhang L. (2017). Application of bag of words model in the prediction of protein subcellular location. *Journal of Food Science and Biotechnology*.

[7] Caporaso N., Whitworth M. B., Fisk I. D. (2018). Protein content prediction in single wheat kernels using hyperspectral imaging. *Food Chemistry*.

[8] Wang X., Li H., Wang R., Zhang Q., Zhang W., Gan Y. (2017). MultiP-apo: a multilabel predictor for identifying subcellular locations of apoptosis proteins. *Computational Intelligence and Neuroscience*.

[9] Nakai K., Kanehisa M. (1991). Expert system for predicting protein localization sites in gram-negative bacteria. *Proteins: Structure, Function, and Genetics*.

[10] Hua S., Sun Z. (2001). Support vector machine approach for protein subcellular localization prediction. *Bioinformatics*.

[11] Du M. Z., Liu S., Zeng Z. (2018). Amino acid compositions contribute to the proteins’ evolution under the influence of their abundances and genomic GC content. *Scientific Reports*.

[12] Nakashima H., Nishikawa K. (1994). Discrimination of intracellular and extracellular proteins using amino acid composition and residue-pair frequencies. *Journal of Molecular Biology*.

[13] Chou K.-C. (2005). Using amphiphilic pseudo amino acid composition to predict enzyme subfamily classes. *Bioinformatics*.

[14] Ju Z., Wang S.-Y. (2018). Prediction of citrullination sites by incorporating *k*-spaced amino acid pairs into Chou’s general pseudo amino acid composition. *Gene*.

[15] Wang T., Yun J., Xie Y., Xiao G. (2017). Finding RNA-protein interaction sites using HMMs. *Hidden Markov Models*.

[16] Zhang S., Duan X. (2018). Prediction of protein subcellular localization with oversampling approach and Chou’s general PseAAC. *Journal of Theoretical Biology*.

[17] Yao Y.-H., Shi Z.-X., Dai Q. (2014). Apoptosis protein subcellular location prediction based on position-specific scoring matrix. *Journal of Computational and Theoretical Nanoscience*.

[18] Zhang S., Liang Y. (2018). Predicting apoptosis protein subcellular localization by integrating auto-cross correlation and PSSM into Chou’s PseAAC. *Journal of Theoretical Biology*.

[19] Liu B., Wang S., Dong Q., Li S., Liu X. (2016). Identification of DNA-binding proteins by combining auto-cross covariance transformation and ensemble learning. *IEEE Transactions on NanoBioscience*.

[20] Liang Y., Zhang S. (2018). Prediction of apoptosis protein’s subcellular localization by fusing two different descriptors based on evolutionary information. *Acta Biotheoretica*.

[21] Lin J., Wang Y. (2011). Using a novel AdaBoost algorithm and Chous pseudo amino acid composition for predicting protein subcellular localization. *Protein & Peptide Letters*.

[22] Bulashevska A., Eils R. (2006). Predicting protein subcellular locations using hierar chical ensemble of bayesian classifiers based on Markov chains. *BMC Bioinformatics*.

[23] Tan Y. T., Rosdi B. A. (2015). FPGA-based hardware accelerator for the prediction of protein secondary class via fuzzy K-nearest neighbors with Lempel-Ziv complexity based distance measure. *Neurocomputing*.

[24] Lai P. T., Huang M. S., Yang T. H. (2018). Statistical principle-based approach for gene and protein related object recognition. *Journal of Cheminformatics*.

[25] Chen Y.-L., Li Q.-Z. (2007). Prediction of the subcellular location of apoptosis proteins. *Journal of Theoretical Biology*.

[26] Kong L., Zhang L. (2019). An ensemble method for multi-type gram-negative bacterial secreted protein prediction by integrating different PSSM-based features. *SAR and QSAR in Environmental Research*.

[27] Yang R., Zhang C., Zhang L., Gao R. (2018). A two-step feature selection method to predict cancerlectins by multiview features and synthetic minority oversampling technique. *BioMed Research International*.

[28] Harsh S., Gaurav R., Alok S. (2015). Probabilistic expression of spatially varied amino acid dimers into general form of Chou’s pseudo amino acid composition for protein fold recognition. *Journal of Theoretical Biology*.

[29] Chen X., Hu X., Yi W., Zou X., Xue W. (2019). Prediction of apoptosis protein subcellular localization with multilayer sparse coding and oversampling approach. *BioMed Research International*.

[30] Yang L. P. (2018). Research of railway fault accident text big data mining key technologies and application.

[31] Li J., Sun L., Yan Q., Li Z., Srisa-an W., Ye H. (2018). Significant permission identification for machine-learning-based android malware detection. *IEEE Transactions on Industrial Informatics*.

[32] Heddam S., Kisi O. (2018). Modelling daily dissolved oxygen concentration using least square support vector machine, multivariate adaptive regression splines and M5 model tree. *Journal of Hydrology*.

[33] Cui X., Yu Z., Yu B., Wang M., Tian B., Ma Q. (2018). UbiSitePred: a novel method for improving the accuracy of ubiquitination sites prediction by using LASSO to select the optimal Chou’s pseudo components. *Chemometrics and Intelligent Laboratory Systems*.

[34] Lópezbegines S., Planabonamaisó A., Méndez A. (2018). Molecular determinants of guanylate cyclase activating protein subcellular distribution in photoreceptor cells of the retina. *Scientific Reports*.

[35] Ruan X. L., Zhou D. M., Nie R. C. (2019). Prediction of apoptosis protein subcellular location based on position-specific scoring matrix and isometric mapping algorithm. *Medical & Biological Engineering & Computing*.

[36] Liang Y., Liu S., Zhang S. (2017). Geary autocorrelation and DCCA coefficient: application to predict apoptosis protein subcellular localization via PSSM. *Physica A: Statistical Mechanics and Its Applications*.

[37] Gu Q., Ding Y.-S., Jiang X.-Y., Zhang T.-L. (2010). Prediction of subcellular location apoptosis proteins with ensemble classifier and feature selection. *Amino Acids*.

[38] Zhou X.-B., Chen C., Li Z.-C., Zou X.-Y. (2008). Improved prediction of subcellular location for apoptosis proteins by the dual-layer support vector machine. *Amino Acids*.

[39] Liang Y. Y., Liu S. Y., Zhang S. L. (2016). Detrended cross-correlation coefficient: application to predict apoptosis protein subcellular localization. *Mathematical Biosciences*.

